# Sox13 and M2-like leukemia-associated macrophages contribute to endogenous IL-34 caused accelerated progression of acute myeloid leukemia

**DOI:** 10.1038/s41419-023-05822-z

**Published:** 2023-05-06

**Authors:** Dongyue Zhang, Xiaoxi Cui, Yifei Li, Rong Wang, Hao Wang, Yibo Dai, Qian Ren, Lina Wang, Guoguang Zheng

**Affiliations:** 1 State Key Laboratory of Experimental Hematology, National Clinical Research Center for Blood Diseases, Haihe Laboratory of Cell Ecosystem, Institute of Hematology & Blood Diseases Hospital, Chinese Academy of Medical Sciences & Peking Union Medical College, 288 Nanjing Road, Tianjin, 300020 China; 2 Tianjin Institutes of Health Science, Tianjin, 301600 China

**Keywords:** Cancer stem cells, Interleukins, Acute myeloid leukaemia, Cancer models, Cancer microenvironment

## Abstract

Interleukin 34 (IL-34) mainly plays physiologic and pathologic roles through the sophisticated multi-ligand signaling system, macrophage colony-stimulating factor (M-CSF, CSF-1)/IL-34-CSF-1R axis, which exhibits functional redundancy, tissue-restriction and diversity. This axis is vital for the survival, differentiation and function of monocytic lineage cells and plays pathologic roles in a broad range of diseases. However, the role of IL-34 in leukemia has not been established. Here MLL-AF9 induced mouse acute myeloid leukemia (AML) model overexpressing IL-34 (MA9-IL-34) was used to explore its role in AML. MA9-IL-34 mice exhibited accelerated disease progression and short survival time with significant subcutaneous infiltration of AML cells. MA9-IL-34 cells showed increased proliferation. In vitro colony forming assays and limiting dilution transplantation experiments demonstrated that MA9-IL-34 cells had elevated leukemia stem cell (LSC) levels. Gene expression microarray analysis revealed a panel of differential expressed genes including Sex-determining region Y (SRY)-box 13 (Sox13). Furthermore, a positive correlation between the expressions of IL-34 and Sox13 was detected human datasets. Knockdown of Sox13 rescued the enhanced proliferation, high LSC level and subcutaneous infiltration in MA9-IL-34 cells. Moreover, more leukemia-associated macrophages (LAMs) were detected in MA9-IL-34 microenvironment. Additionally, those LAMs showed M2-like phenotype since they expressed high level of M2-associated genes and had attenuated phagocytic potential, suggesting that LAMs should also contribute to IL-34 caused adverse phenotypes. Therefore, our findings uncover the intrinsic and microenvironmental mechanisms of IL-34 in AML and broadens the knowledge of M-CSF/IL-34-CSF-1R axis in malignancies.

## Introduction

Acute myeloid leukemia (AML) is the most common type of acute leukemia in adults [1]. Although clinical treatment has been greatly improved, most AML patients eventually experience relapse and death. As a highly heterogeneous disease, both intrinsic and microenvironmental abnormalities participate in the transformation, progression and relapse of AML [2–4]. Elucidating the mechanisms causing adverse phenotypes of AML including exuberant proliferation [5], more leukemia stem cells (LSCs) [6, 7], massive extramedullary dissemination [2], etc. will provide clues for better outcome.

Interleukin-34 (IL-34) was first identified as another ligand for colony stimulating factor-1 (CSF-1, also known as macrophage colony-stimulating factor, M-CSF) receptor (CSF-1R, CD115) [8]. Although protein-tyrosine phosphatase (PTP)-ζ and Syndecan-1 are also identified as IL-34 receptors [9], much attention has been paid to CSF-1R [10]. Binding of IL-34 to CSF-1R promotes the proliferation and differentiation of monocytes and macrophages [8, 11], whereas IL-34 deficiency results in the decrease of Langerhans cells and microglias but not monocytes or macrophages [12] owing to the redundancy in the M-CSF/IL-34-CSF-1R axis. This axis also plays pathologic roles in a broad range of diseases [13]. Abnormal expression of M-CSF isoforms, resulted from alternative splicing and posttranslational modifications [14], and IL-34 was reported in various types of malignancies [15–17]. Both intrinsic and microenvironmental mechanisms have been proposed for this axis in malignancies. Specifically, high level IL-34 not only promotes the proliferation, invasion and chemoresistance of cancer cells [16, 17], but also reprograms tumor associated macrophages (TAMs) to acquire a specific phenotype affecting tumor progression [18]. However, IL-34 lacks sequence similarity with M-CSF [11]. M-CSF and IL-34 have differences at steady state and in diseases [13, 19]. Even different isoforms of M-CSF may have diverse pathologic roles in leukemia [20, 21]. These facts reveal the complexity of this axis. Until now, the role of IL-34 in leukemia, especially on LSCs, remains unknown.

Sex-determining region Y (SRY)-box 13 (Sox13), a member of the SRY-related high mobility group (HMG) box (SOX) proteins, plays essential roles in embryonic development, cell fate decision and cancer development [22]. Sox13 promotes the proliferation, migration and metastasis of cancer cells through different mechanisms [23–26]. Recent evidence demonstrated that Sox13 maintained stem-like properties in hepatocellular carcinoma [27]. However, little is known about its pathologic roles in blood diseases. Furthermore, the link between IL-34 and Sox13 have not been established.

In this study, MLL-AF9 induced mouse AML model overexpressing IL-34 was used to explore the role of IL-34 in AML. Overexpression of IL-34 in AML cells accelerates AML progression by promoting cell proliferation and elevating LSC frequency. Furthermore, Sox13 contributes to the pro-leukemic effects in AML cells overexpressing IL-34. Moreover, increased infiltration of M2-like leukemia-associated macrophages (LAMs) with attenuated phagocytic potential may also contribute to the accelerated progression.

## Materials and methods

### Mouse AML models

**Fig. 1:**
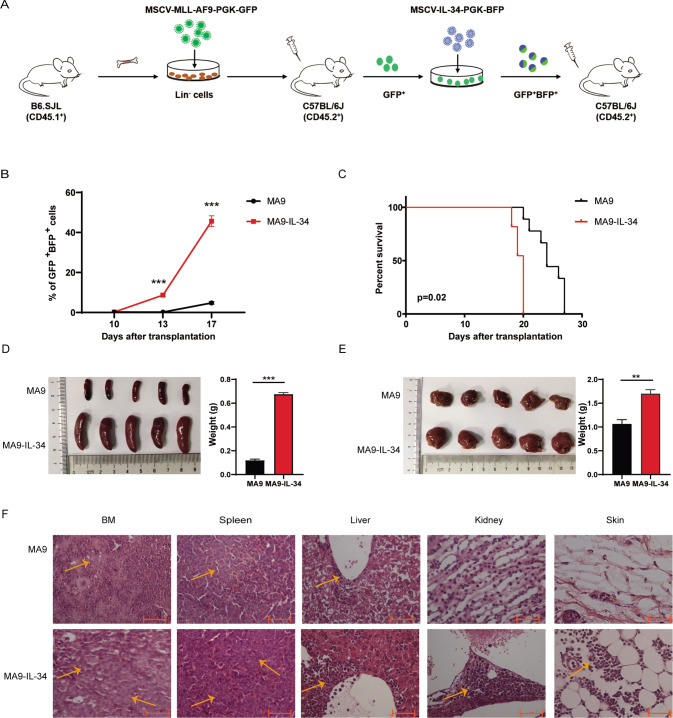
Overexpression of IL-34 accelerated AML progression. **A** Schematic overview showing the establishment of the MA9 and MA9-IL-34 mouse AML models. **B**–**F** 1 × 10^5^ GFP^+^BFP^+^ cells were transplanted into recipient mice and the progression of AML was studied. **B** The PB leukemia cell levels were monitored at indicated time points (*n* = 4). **C** The survival of AML mice is shown in Kaplan-Meier curves (MA9, *n* = 10; MA9-IL-34, *n* = 11). **D**–**E** The size and weight of spleens (**D**) and livers (**E**) on day 17 are shown. **F** The HE-stained sections of BM, spleen, liver, kidney and skin were examined under a light microscope. Scale bars: 50μm. Data are presented as mean ± S.E.M. Unpaired Student’s *t* test and Kaplan–Meier estimates were used. **p < 0.01, ***p < 0.001.

The establishment of mouse AML model overexpressing IL-34 is shown in Fig. 1A. Briefly, GFP^+^ AML cells were sorted from MLL-AF9 induced AML mice [28, 29] and infected with blank (pMSCV-PGK-BFP) or IL-34 (pMSCV-IL-34-PGK-BFP) retrovirus. The GFP^+^BFP^+^ leukemia cells, named MA9 and MA9-IL-34 cells, were sorted and transplanted into C57BL/6 J mice by tail intravenous injection to establish MA9 and MA9-IL-34 mice.

To knockdown (KD) Sox13 in MA9-IL-34 cells, validated small hairpin (shRNA) lentivirus targeting mouse Sox13 (the sequences is 5’CCGGGGATGTCAAAGGGACC CAAGACTCGAGTCTTGGGTCCCTTTGACATCCTTTTTG3’) was constructed using pLKO.1-puro-mcherry vector by IBSBIO technology Inc. MA9-IL-34 cells were infected with scramble (pLKO.1-S-sc) or KD (pLKO.1-S-sh1) lentivirus. The GFP^+^BFP^+^RFP^+^ cells, named IL-34-S-sc and IL-34-S-sh1 cells, were sorted and transplanted into C57BL/6 J mice to establish IL-34-S-sc and IL-34-S-sh1 mice.

### Cell cycle and apoptosis assays

In Ki-67 assays, 1 × 10^6^ AML cells were sorted, fixed and permeabilized by Cytofix/Cytoperm™ Fixation/Permeabilization Solution Kit (BD, San Jose, CA). Then, the cells were stained with PE-conjugated Ki-67 for 30 min. Hoechst 33342 was added before flow cytometry analysis.

In BrdU incorporation assays, AML mice were intraperitoneally injected with 200 μl BrdU (10 mg/ml). 3 × 10^6^ AML cells were sorted 16 h later and stained with a Pharmingen TM APC BrdU Flow Kit (BD, San Jose, CA) following the manufacturer’s protocols before flow cytometry analysis.

In apoptosis assays, single cell suspension was prepared with Annexin V binding buffer and incubated with APC-conjugated Annexin V antibody for 15 min in the dark. Then, PI (500 μg/ml) was added and cells were detected by flow cytometry.

### Colony forming assay

Cells were sorted and resuspended in M3434 complete medium. Five hundred cells in 500 μl were plated into 24-well plate. After seven days, colonies were scanned and counted by a high-content analysis system (PerkinElmer, UK). The colonies were divided into three types according to their morphology: the type A colonies have a dense center and smooth edge; the type B colonies have a compact center and unsmooth edge with migrating cells; the type C colonies have relatively loose structure.

### Limiting-dilution transplantation

After sorting, 5 × 10^2^ to 5 × 10^4^ MA9 or MA9-IL-34 cells were transplanted into C57BL/6 J mice. The survival of the mice was recorded. The frequency of LSCs was calculated using extreme limiting dilution analysis (ELDA) online software.

### Gene expression microarray

The MA9-c-Kit^-^, MA9-c-Kit^+^ and MA9-IL-34 AML cells were sorted by flow cytometry. Microarrays were completed following standard protocols in Shanghai Majorbio Biopharm Technology (China). Standard analyses were performed by the online Majorbio Cloud Platform. Fold change (FC) ≥ 2.0 and *p* adjust <0.05 were used as the cutoff for screening differentially expressed genes (DEGs).

### Statistical analysis

All experiments were repeated two to three times. The results were represented as means ± S.E.M. GraphPad Prism 8.0 (GraphPad Software, CA) were used for data analysis. When parameters followed Gaussian distribution, unpaired Student’s *t* test was used for comparisons between two groups, whereas one-way ANOVA was used for comparisons among multiple groups. Kaplan–Meier estimates were used for survival curves. ELDA was used for the limiting-dilution transplantation experiments. *P* < 0.05 was considered statistically significant.

## Results

### MA9-IL-34 cells cause accelerated AML progression

To study the role of IL-34 in AML progression, the MLL-AF9 induced AML model overexpressing IL-34 was established (Fig. 1A). The expression of IL-34 was verified by qRT-PCR, Western blot and ELISA (Figs. S1A-C, S2A-B). Both MA9 and MA9-IL-34 cells were GFP^+^BFP^+^CD3^-^CD19^-^CD11b^+^Gr-1^+^ (Fig. S1D). The expression of IL-34 receptors was also studied. CD115 was detectable at mRNA and protein levels, Syndecan-1 was detectable at mRNA level whereas PTP-ζ was undetectable (Fig. S1E, F). Wright staining showed that MA9-IL-34 cells had larger nucleoli, less but darker cytoplasm than MA9 cells (Figure S1G). MA9-IL-34 mice exhibited higher levels of PB leukemia cells since day 13 (Fig. 1B) and shorter survival times than MA9 mice (Fig. 1C). Hepatosplenomegaly was more severe in MA9-IL-34 mice than MA9 mice on day 17 (Fig. 1D, E). Furthermore, pathologic analysis showed that more infiltrating AML cells were observed in tissues from MA9-IL-34 mice than MA9 mice. Interestingly, subcutaneous infiltration of AML cells was observed in MA9-IL-34 mice (Fig. 1F, Fig. S1H). These results suggested that overexpression of IL-34 in AML cells accelerated AML progression.

### Characteristics of MA9-IL-34 cells

**Fig. 2:**
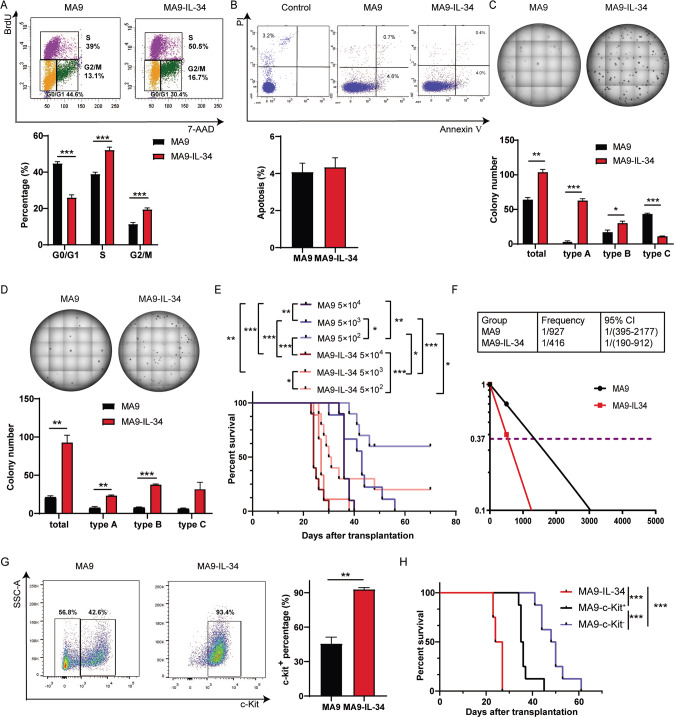
The characteristics of MA9-IL-34 cells. **A**–**D** The mice were transplanted with MA9 or MA9-IL-34 cells on day 0 and sacrificed on day 16. **A** Mice were sacrificed 16 h after intraperitoneal injection of 200 μg BrdU. Then assays were performed following standard protocols. The representative flow cytometric results are shown (upper), and the percentages of G0/G1-, S- and G2/M-phase AML cells are plotted (lower). **B** Annexin V and PI staining was performed following standard protocols. The representative flow cytometric results are shown (upper), and the percentage of apoptotic AML cells is plotted (lower). **C**–**D** Primary (**C**) and secondary (**D**) colony forming assays were performed when 500 AML cells in M3434 medium were seeded each well into 24-well plates and cultured for 7d. The representative results are shown (upper), and colony numbers are plotted (lower). **E**, **F** Different numbers of sorted AML cells (5 × 10^4^, 5 × 10^3^, 5 × 10^2^ for each group) were transplanted into recipient mice (*n* = 9 to 10 for each group). The survival of mice is shown in Kaplan–Meier curves (**E**). The frequency of LSCs was calculated using ELDA software (**F**). **G** AML cells were stained with c-Kit. The representative flow cytometric results are shown (left), and the percentage of c-Kit^+^ cells is plotted (right). **H** Equal numbers of sorted MA9-c-Kit^-^, MA9-c-Kit^+^ and MA9-IL-34 cells were transplanted into recipient mice. The survival of mice is shown in Kaplan–Meier curves (*n* = 8 for each group). Data are presented as mean ± S.E.M. Unpaired Student’s *t* test, one-way ANOVA tests and Kaplan–Meier estimates were used. *p < 0.05, **p < 0.01, ***p < 0.001.

To explore the mechanism leading to the accelerated AML progression, the characteristics of AML cells were first studied. BrdU assay showed that more S and G2/M phase but fewer G0/G1 phase cells were detected in MA9-IL-34 cells than MA9 cells (Fig. 2A). The apoptotic rate between two groups had no significant difference (Fig. 2B). Colony forming potential partly reflects the LSC level, which is associated with poor prognosis in AML. The in vitro colony forming experiments demonstrated that MA9-IL-34 cells formed more colonies than MA9 cells in both primary and secondary plating experiments (Fig. 2C, D). There are three types of colonies (Figure S3) [3, 30]. MA9-IL-34 cells formed more type A and type B colonies than MA9 cells in primary plating experiment. Similar results were also obtained in secondary plating experiments (Fig. 2C, D). Limiting dilution transplantation experiments were used to analyze LSC frequency. 5 × 10^4^ or 5 × 10^3^ cells caused 100% death in both groups. 5 × 10^2^ cells caused 80% death in MA9-IL-34 group but 40% in MA9 group (Fig. 2E). The LSC level in MA9-IL-34 cells was approximately 2-fold higher than that in MA9 cells (Fig. 2F). The expression of c-Kit, an important marker of LSCs, was detected by flow cytometry. More than 90% MA9-IL-34 cells whereas approximately half MA9 cells were c-Kit^+^ (Fig. 2G). Equal numbers of MA9-c-Kit^-^, MA9-c-Kit^+^ and MA9-IL-34 cells were sorted and transplanted into recipient mice. As expected, MA9-IL-34 mice had the shortest survival time (Fig. 2H). Taken together, AML cells overexpressing IL-34 have enhanced proliferation and elevated LSC frequency, which contribute to the accelerated AML progression.

### Identification of intrinsic molecules contributing to the pro-leukemic effects in MA9-IL-34 cells

**Fig. 3:**
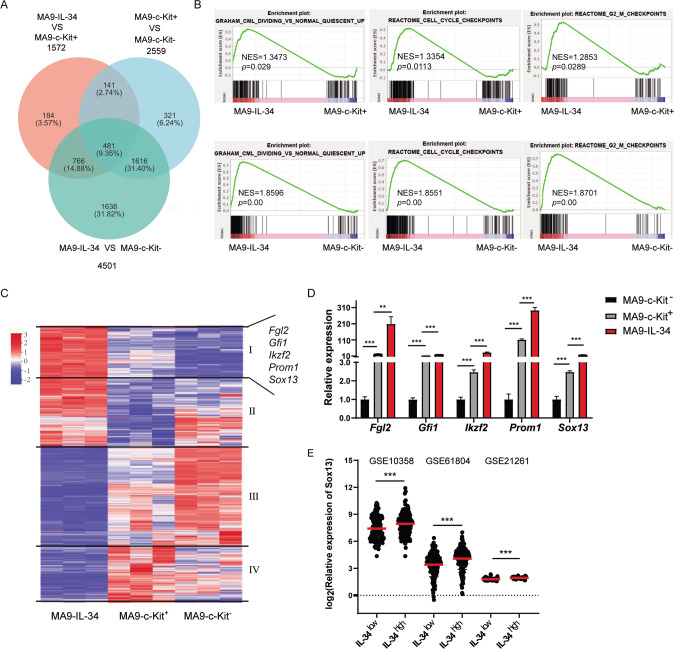
Identification of intrinsic molecules contributing to the pro-leukemic effects in MA9-IL-34 cells. MA9-c-Kit^-^, MA9-c-Kit^+^, MA9-IL-34 cells were sorted and microarray was performed. **A** Venn diagram shows overlaps of DEGs obtained from each pair. **B** GSEA analysis shows the enrichment of genes in annotations between MA9-IL-34 and MA9-c-Kit^+^/MA9-c-Kit^-^ groups. **C** Heat map shows the expression levels of 1572 genes, which are the DEGs between MA9-IL-34 and MA9-c-Kit^+^ groups, in three groups. Those genes are subdivided into 4 clusters based on the expression patterns in three groups. **D** The expression of selected genes was validated by qRT-PCR. **E** The expression level of Sox13 in the GSE10358 (*n* = 304), GSE61804 (*n* = 324) and GSE21261 (*n* = 76) is shown. For each dataset, AML cases were divided into IL-34^low^ and IL-34^high^ groups, and the corresponding expression of Sox13 is plotted. Data are presented as mean ± S.E.M. Unpaired Student’s *t* test, one-way ANOVA tests were used. **p < 0.01, ***p < 0.001.

MA9-c-Kit^−^, MA9-c-Kit^+^ and MA9-IL-34 cells were undergone expression microarray to screen intrinsic key molecules contributing to the accelerated disease progression. A Venn diagram shows the numbers of DEGs between different pairs of the samples (Fig. 3A). The number of DEGs between MA9-IL-34 and MA9-c-Kit^-^ was greater than that between MA9-IL-34 and MA9-c-Kit^+^, which was in accordance with functional observations. GSEA analysis showed that the genes in annotations “quiescent up”, “cell cycle checkpoints” and “G2/M checkpoints” were enriched in MA9-IL-34 cells versus MA9-c-Kit^+^ or MA9-c-Kit^-^ cells (Fig. 3B). Furthermore, genes in annotations related to worse phenotypes in tumors were enriched in MA9-IL-34 cells versus MA9-c-Kit^+^/MA9-c-Kit^-^ (Fig. S4A) or MA9-c-Kit^+^ cells (Fig. S4B). Moreover, embryonic stem cell core associated genes were enriched in MA9-IL-34 cells versus MA9-c-Kit^-^ cells (Fig. S4C). The 1572 genes (DEGs between MA9-IL-34 and MA9-c-Kit^+^ cells) were divided into 4 clusters based on their expression pattern in three groups. The cluster I with 428 genes was of interest (Fig. 3C). Then, 126 genes were first selected since they were also DEGs between MA9-c-Kit^+^ and MA9-c-Kit^-^ groups, furthermore the expression was higher in MA9-c-Kit^+^ cells than MA9-c-Kit^-^ cells. Five genes with high expression levels, which related to stemness of tumor cells, were selected and further verified by qRT-PCR (Fig. 3D). Notably, a significant positive correlation between the expression of *IL-34* and *SOX13* was detected in the human datasets GSE10358, GSE21261 and GSE61804 (Fig. 3E). Hence, Sox13 was selected for further study.

### Sox13 contributes to the pro-leukemic effects in MA9-IL-34 cells

**Fig. 4:**
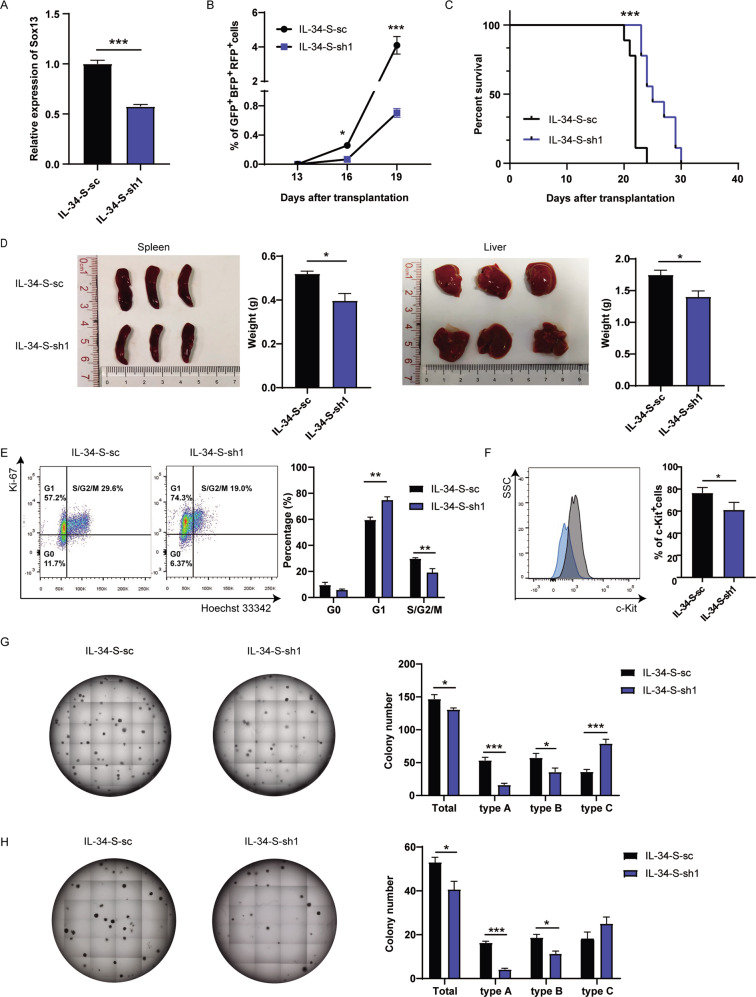
Sox13 contributes to the pro-leukemic effects in MA9-IL-34 cells. The expression of Sox13 was knockdown in MA9-IL-34 cells by shRNA strategy. **A** The expression of Sox13 was detected by qRT-PCR. **B**–**D** Equal numbers of IL-34-S-sc or IL-34-S-sh1 cells were sorted and transplanted into recipient mice. **B** The PB AML cells were monitored at indicated time points (*n* = 4). **C** The survival of mice is shown in Kaplan–Meier curves (*n* = 9 for each group). **D** The sizes and weights of spleens and livers on day 19 are shown. **E** IL-34-S-sc or IL-34-S-sh1 cells were stained with Ki-67 and Hoechst 33342. The representative flow cytometric results are shown (left), and the percentages of G0-, G1-, and S/G2/M-phase AML cells are plotted (right). **F** GFP^+^BFP^+^RFP^+^ cells were stained with c-Kit. The representative flow cytometric results are shown (left), and the percentages of c-Kit^+^ cells are plotted. **G**, **H** Primary (**G**) and secondary (**H**) colony forming assays were performed when 500 AML cells in M3434 medium were seeded each well into 24-well plates and cultured for 7d. The representative results are shown (left), and the colony numbers are plotted (right). Data are presented as mean ± S.E.M. Unpaired Student’s *t* test, one-way ANOVA tests and Kaplan–Meier estimates were used. *p < 0.05, **p < 0.01, ***p < 0.001.

To investigate whether Sox13 contributed to the malignant phenotype, MA9-IL-34 cells were infected with pLKO.1-S-sc or pLKO.1-S-sh1 to construct IL-34-S-sc and IL-34-S-sh1 mouse AML models. The knockdown efficiency of *Sox13* was verified by qRT-PCR (Fig. 4A). IL-34-S-sc and IL-34-S-sh1 cells were both GFP^+^BFP^+^RFP^+^CD3^-^CD19^-^Gr-1^+^CD11b^+^ (Fig. S5A, B). The IL-34-S-sh1 mice exhibited lower PB leukemia cell levels since day 16 after transplantation (Fig. 4B) and had longer survival times than IL-34-S-sc mice (Fig. 4C). Hepatosplenomegaly was milder (Fig. 4D) and fewer infiltrating AML cells (Fig. S5C) were detected in liver in IL-34-S-sh1 mice than IL-34-S-sc mice on day 19. Pathologic analysis showed that subcutaneous infiltration of AML cells was observed in IL-34-S-sc mice but not IL-34-S-sh1 mice although leukostasis was observed in subcutaneous blood vessels in IL-34-S-sh1 mice (Fig. S5D). Ki-67 experiments showed that more G1 phase cells and fewer S/G2/M phase cells were detected in IL-34-S-sh1 cells (Fig. 4E). Knockdown of *Sox13* resulted in the decreased expression of c-Kit (Fig. 4F). Furthermore, in vitro colony forming assay showed that IL-34-S-sh1 cells formed fewer colonies than IL-34-S-sc cells in primary and secondary plating experiments (Fig. 4G, H). Taken together, these results suggest that Sox13 acts as intrinsic factor contributing to the pro-leukemic effects in AML mice overexpressing IL-34.

### LAMs contribute to the pro-leukemic effects in MA9-IL-34 mice

**Fig. 5:**
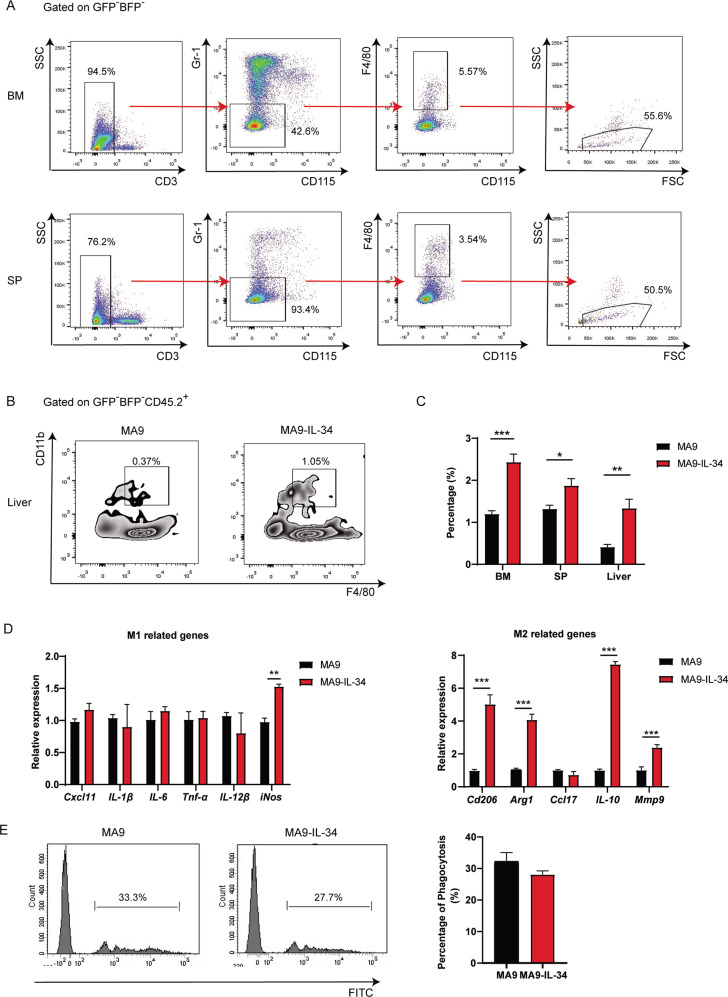
LAMs contribute to the pro-leukemic effects in MA9-IL-34 mice. AML mice were sacrificed at middle stage of leukemia. BM and SP samples were prepared. **A** Gating strategy for LAMs from BM and SP samples by flow cytometry. **B** Gating strategy for LAMs from liver samples by flow cytometry. **C** The frequencies of LAMs in different tissues are plotted. **D**, **E** LAMs were sorted from BM samples. The expression of M1- and M2- associated genes in LAMs was detected by qRT-PCR (**D**). The representative flow cytometric results (left) and the positive rates (right) of in vitro uptake experiments to assess the phagocytotic potential of LAMs are shown (**E**). Data are presented as mean ± S.E.M. Unpaired Student’s *t* test, one-way ANOVA tests were used. *p < 0.05, **p < 0.01, ***p < 0.001.

Immunologic microenvironment plays important roles in leukemia progression [31, 32]. No significant difference was detected on the distribution of B cells, NK cells, CD4^+^ cells and CD8^+^ T cells in spleen samples as well as granulocytes in BM samples (Fig. S6 A–D). Signaling through CSF-1R, the major receptor of IL-34, is vital for the differentiation and survival of mononuclear phagocytes; and macrophages play important roles in malignancies including leukemia [33–36]. The macrophages in BM and spleen were gated as the SSC^int/lo^ subpopulation in the CD3^-^Gr-1^lo^F4/80^+^CD115^int^ population while those in liver were gated as the CD11b^+^F4/80^+^CD45^+^ population [36] (Fig. 5A, B). Higher levels of LAMs were detected in those tissues in MA9-IL-34 mice than MA9 mice (Fig. 5C). Furthermore, it is well accepted that M1 macrophages have anti-tumor effects whereas M2 macrophages have pro-tumor effects [34]. LAMs from MA9-IL-34 mice expressed higher levels of M2-associated genes including *Cd206*, *Arg1*, *Il-10* and *Mmp9* than MA9 mice, whereas expressed similar levels of M1-associated genes as MA9 mice (Fig. 5D). We used a multiple factor weight comprehensive analysis to define the activation phenotype of LAMs [33, 36]. The results showed that LAMs in MA9-IL-34 microenvironment have more M2 characteristics (Fig. S6E), or simply called as M2-like LAMs. Moreover, phagocytosis is an important anti-tumor function of macrophages [21, 37, 38]. LAMs in MA9-IL-34 mice had lower phagocytic potential than those in MA9 mice (Fig. 5E). Taken together, there are more M2-like LAMs with lower phagocytic potential in MA9-IL-34 microenvironment, which suggest that LAM-associated microenvironmental mechanisms should also contribute to the pro-leukemic effects in MA9-IL-34 mice.

### Pexidartinib partly attenuates AML progression in MA9-IL-34 mice

Pexidartinib, the CSF-1R inhibitor, was used to test the effects in MA9-IL-34 mice (Fig. S7A). Pexidartinib decreased the weight of spleen and liver, in MA9-IL-34 mice (Fig. S7B). Additionally, it suppressed subcutaneous infiltration of AML cells in MA9-IL-34 mice (Fig. S7C). Moreover, it significantly lowered the AML cell burden in BM and SP (Fig. S7D). Furthermore, it downregulated the expression of c-kit in MA9-IL-34 cells (Fig. S7E). Besides, it lowered the level of LAMs in BM and spleen in MA9-IL-34 mice (Fig. S7F). Prolong in survival time was not observed (Fig. S7G), which may be partly due to rapid progression of MLL-AF9-induced AML model and incomplete blockage of excessive IL-34 by the recommended dosage of Pexidartinib. Therefore, M-CSFR inhibitor partly attenuated the progression of MA9-IL-34 mice.

## Discussion

The development of malignancies is regulated by complex networks comprising both intrinsic and microenvironmental factors. The effect of M-CSF/IL-34-CSF-1R axis in malignancies is a good example since it covers both aspects. In fact, abnormal expression and function of both ligands and receptor were reported in various malignancies including pre-leukemia, leukemia and lymphoid malignancies [10, 17, 39]. Furthermore, abnormal activation of this axis may directly stimulate malignant cells and/or shape immunologic microenvironment [15, 40, 41]. Notably, this axis is vital for monocytic lineage cells, either normal or malignant. Therefore, acute myelomonocytic leukemia, such as the MLL-rearranged AML [42], is a good model to elucidate the mechanism of this axis in malignancies. However, the multiple-ligand axis exhibits expressional and functional diversities under both physiological and pathological conditions. IL-34 is regarded as a tissue-restricted ligand of CSF-1R in skin and central nerve system [12]. Furthermore, M-CSF isoforms, i.e. secretory (sM-CSF), membrane-bound (mM-CSF), and extracellular matrix or proteoglycan (PG-M-CSF), exhibit different expression patterns and functions [43]. While sM-CSF elicits short-term signal and exerts short- or long-distance effects by autocrine, paracrine or endocrine mechanisms, mM-CSF provides persistent activation signal and regulates physically contacted cells by juxtacrine mechanism [43, 44]. We previously demonstrated that macrophages shaped by sM-CSF and mM-CSF showed significant phenotypic diversity; and mM-CSF but not sM-CSF prolonged the survival of AML mice by inducing the differentiation of AML cells and polarizing LAMs to have high phagocytotic potential [20, 21]. Here we report that overexpression of IL-34 in AML cells results in accelerated AML progression, short survival and significant subcutaneous infiltration. Furthermore, Sox13-associated upregulated proliferation and LSC level account for intrinsic mechanism while M2-like LAMs account for microenvironmental mechanism. Hence, our study gives new insight into the pathologic role of IL-34 in AML and broadens the knowledge of the M-CSF/IL-34-CSF-1R axis in malignancies.

The survival and function of cells are tightly regulated by a complex network and a single gene abnormality in malignant cells may frequently lead to several adverse phenotypes [3, 30, 45]. Overexpression of IL-34 in AML cells simultaneously causes enhanced malignant proliferation, more LSCs and significant subcutaneous infiltration. MLL-AF9 induced AML is a good model for monocytic lineage malignancies [28] and the M-CSF/IL-34-CSF-1R axis provides signals to boost malignant proliferation. Overexpression of IL-34 in human MLL-rearranged cell lines, THP1 and Moml-13, also have pro-proliferative effect [46]. In fact, IL-34 is abnormally expressed and promotes malignant proliferation in different types of cancers [16, 17, 47, 48]. Therefore, although the physiologic significance of IL-34 seems to be tissue-restricted, abnormally expressed IL-34 can provide pro-proliferative signals in a broad range of malignancies.

LSCs are responsible for the initiation, drug resistance, relapse and bad outcome in leukemia [7, 49]. A novel finding of this study is that overexpression of IL-34 in AML cells increases LSC frequency. Nowadays, there is not a good method to mark LSCs although the mouse L-GMP, the typical markers of which is IL-7R^-^Lin^-^Sca-1^-^c-Kit^+^CD34^+^FcgRII/III^+^, is suggested [50]. However, L-GMP frequency is not suitable for this particular model [3, 30] since considerable S-phase L-GMPs were detected either in reports [45] or from our observations, demonstrating that at least part of L-GMPs are proliferative instead of quiescent. Furthermore, most AML cells in this model were Gr-1^+^ but not the Lin^-^ cells so that L-GMP in MA9-IL-34 cells can hardly be gated and the G0/G1 phase L-GMP level is lower than LSC level obtained from functional experiments. Most importantly, the conclusion is obtained based on the functional experiments, i.e. the colony forming and limiting dilution transplantation experiments. There may be another question, i.e. enhanced proliferation and high LSC level seem to be controversial because LSCs/tumor stem cells (TSCs) are normally considered as quiescent cells [51, 52]. In fact, malignant cells are composed of heterogeneously hierarchical sub-populations [53]. In this case, AML cells can be simply classified into LSCs (small number) and non-LSCs (abundant). Some key molecules may simultaneously participate in the maintenance of LSCs and the proliferation of non-LSCs. In fact, malignancies with both adverse characteristics are frequently observed not only in research models but also in clinical patients [3, 30, 54]. How IL-34 boosts LSCs has not been fully understood although we demonstrate that Sox13 is partly responsible for this.

Subcutaneous infiltration is another pathologic characteristic of MA9-IL-34 cells. It’s worth noting that the significant physiologic effects of IL-34 seem to be restricted in skin and central nerve system [12]. However, enhanced intracerebral invasion has not been observed possibly due to the blood brain barrier. Nevertheless, extramedullary infiltration of leukemia cells has long been observed and considered as a bad marker for leukemia patients [2]. Patients suffer combined extramedullary and BM relapse has worse prognosis than those with BM relapse alone [55]. Furthermore, leukemia cutis as a specific extramedullary infiltration signifies a poor prognosis of AML [56, 57]. Therefore, although the mechanism is not clear, this phenomenon accounts for another adverse effect of IL-34 in AML.

The association between IL-34 and Sox13 has not been previously reported. Sox13 at least partly mediates the adverse phenotypes including enhanced proliferation, more LSCs and subcutaneous infiltration in MA9-IL-34 cells. The pro-proliferative effects of Sox13 in solid tumors have been reported and different pathways, such as the Wnt-β-catenin pathway, the SOX13-TRIM11-YAP axis as well as the PAX8-Aurora B/Cyclin B1 pathway, have been proposed [24–26]. Recent observation showed that Sox13 regulated cancer stem-like properties in hepatocellular carcinoma cells but the mechanism was not clear [27]. Therefore, although the pathway from IL-34 to Sox13 and downstream adverse phenotypes has been observed in AML, the molecular mechanisms including how IL-34 regulates the expression of Sox13, the downstream events of Sox13 leading to enhanced proliferation, more LSCs and subcutaneous infiltration have not been elucidated and deserve further exploration.

Macrophages are an important component of the physiologic and pathologic microenvironment and play sophisticated roles in the initiation and progression of malignancies [33, 35, 58]. Although the M1/M2 criteria has obvious limitation, the evidence demonstrating the anti-tumor M1 or M1-like macrophages and the pro-tumor M2 or M2-like macrophages has been well documented [34]. Furthermore, the M-CSF/IL-34-CSF-1R axis is vital for the survival and function of macrophages although different ligand may have diverse effects [19–21, 41]. Here we report that increased LAMs are detected in MA9-IL-34 microenvironment. Whether this phenomenon is caused by increased recruitment and/or local expansion is not clear. Furthermore, those LAMs exhibit more M2 characteristics since they express high level of M2 phenotype associated genes including *Cd206*, *Arg1*, *Il-10* and *Mmp9*, which imply that they may have pro-leukemic effects. Moreover, LAMs in MA9-IL-34 microenvironment exhibit low phagocytic potential, which mediates the anti-tumor effects of macrophages [37, 38]. Thus, more M2-like LAMs are polarized in the MA9-IL-34 microenvironment and at least partly account for the accelerated AML progression.

In conclusion, we demonstrate that IL-34 overexpressed in AML cells accelerates AML progression by affecting both AML cell themselves and LAMs in the leukemia microenvironment. Upregulated Sox13 accounts for the promoted proliferation, increased LSC level and subcutaneous infiltration.

## Supplementary information

Figure S1

Figure S2

Figure S3

Figure S4

Figure S5

Figure S6

Figure S7

Supplemental materials and methods

Supplemental table

checklist

## Data Availability

Bulk RNA-seq data are available in the National Center for Biotechnology Information Gene Expression Omnibus database under accession number GSE213201.
