# Supplementary figures and images for: Sox13 and M2-like leukemia-associated macrophages contribute to endogenous IL-34 caused accelerated progression of acute myeloid leukemia
Source: Cell Death Dis. 2023 May 6;14(5):308. doi: 10.1038/s41419-023-05822-z (PMC10164149; doi:10.1038/s41419-023-05822-z)

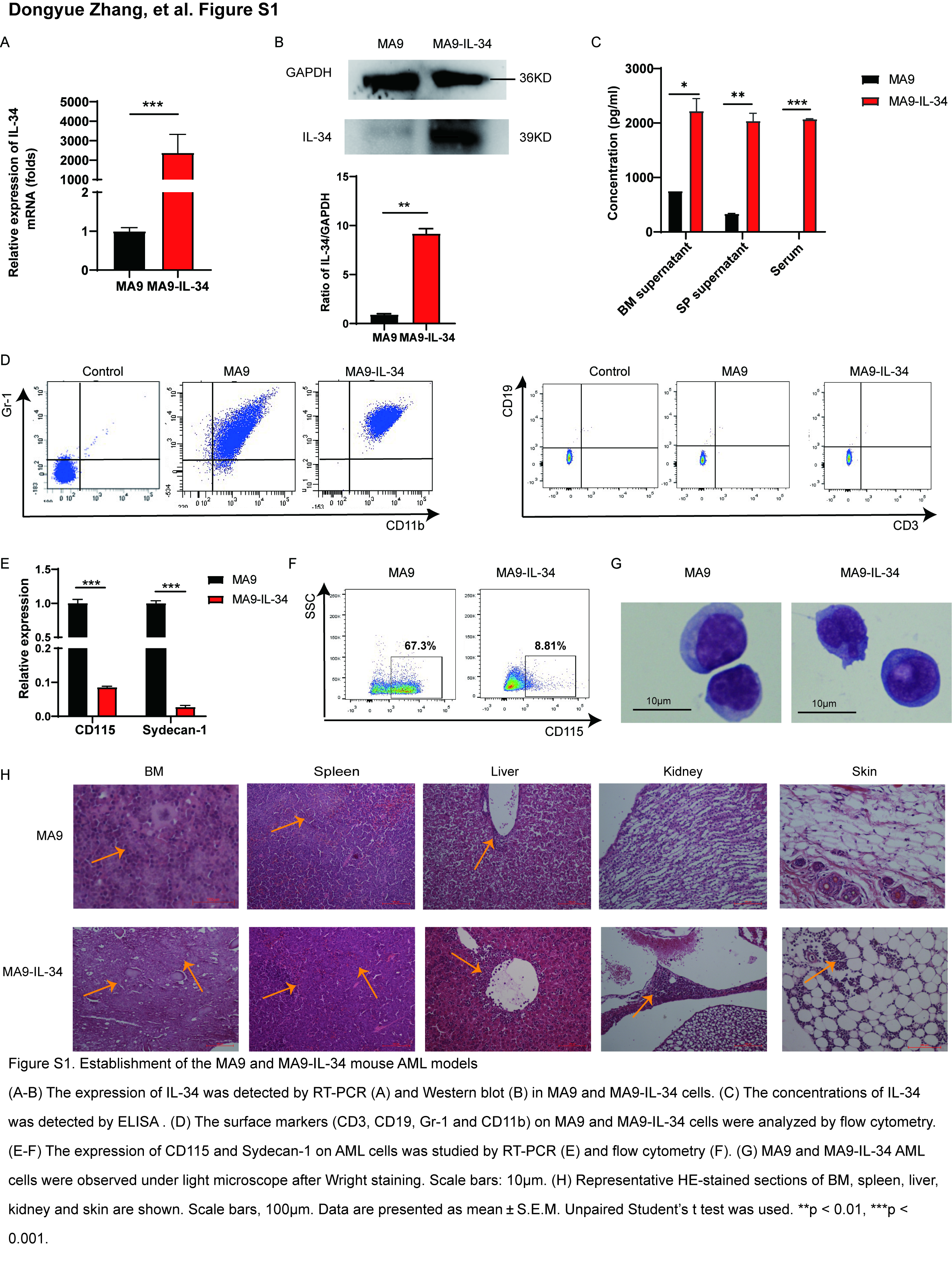

Supplement: Supplementary file 1 — Figure S1 [file 41419_2023_5822_MOESM1_ESM.tif]

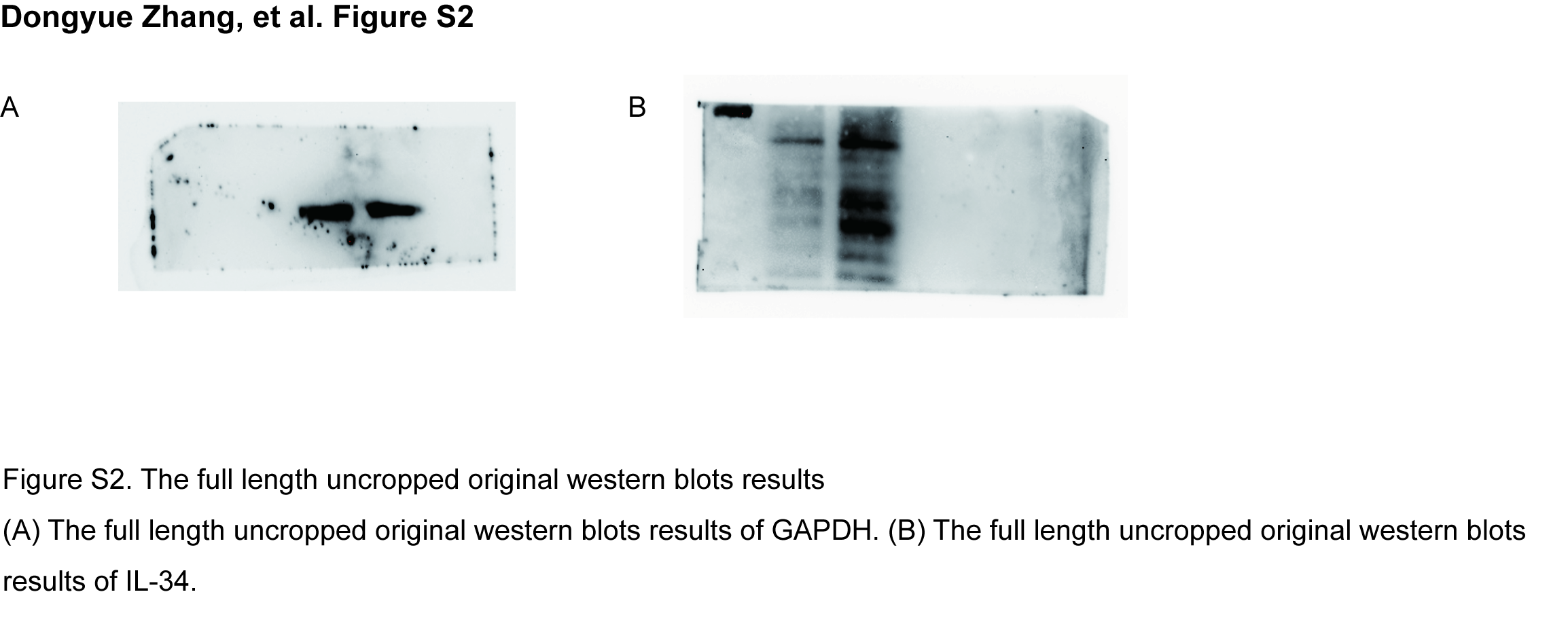

Supplement: Supplementary file 2 — Figure S2 [file 41419_2023_5822_MOESM2_ESM.tif]

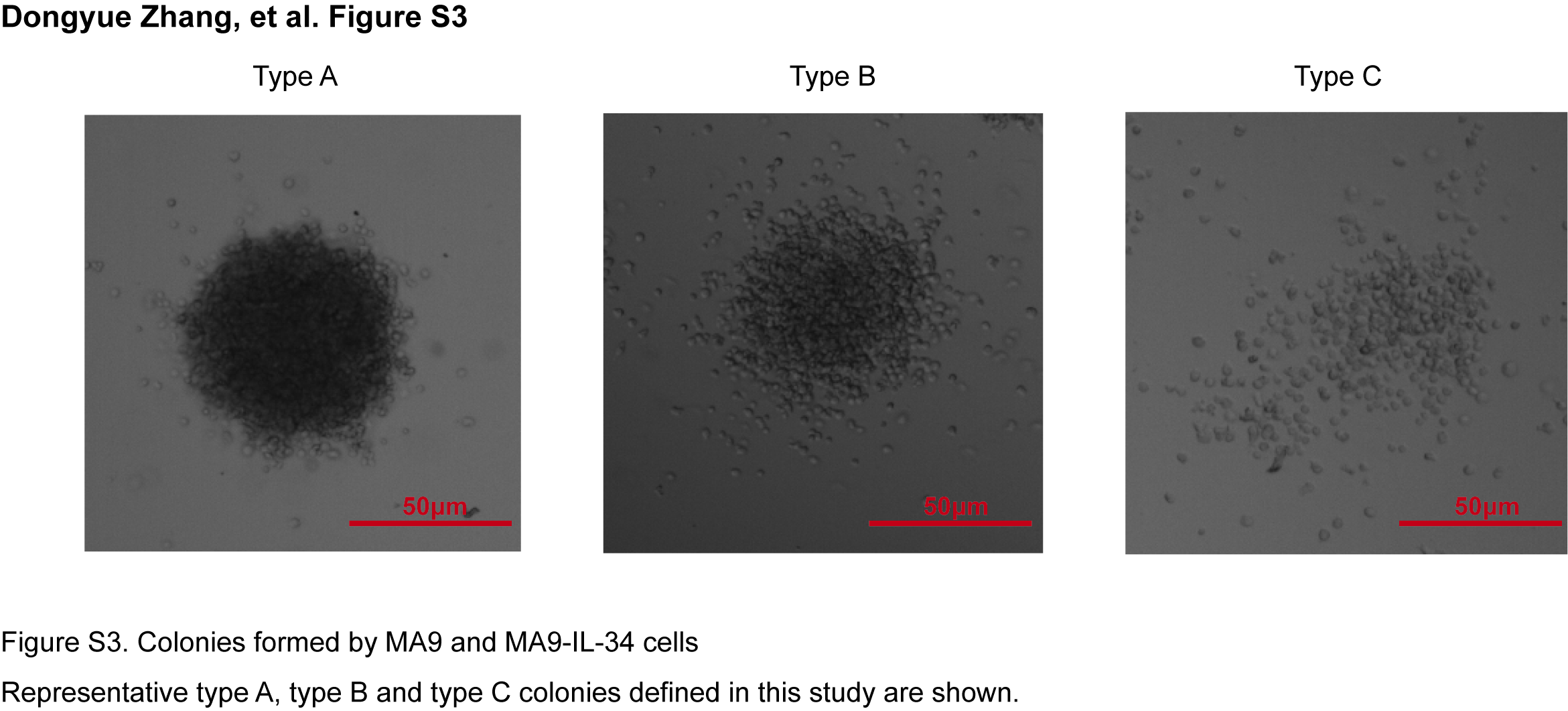

Supplement: Supplementary file 3 — Figure S3 [file 41419_2023_5822_MOESM3_ESM.tif]

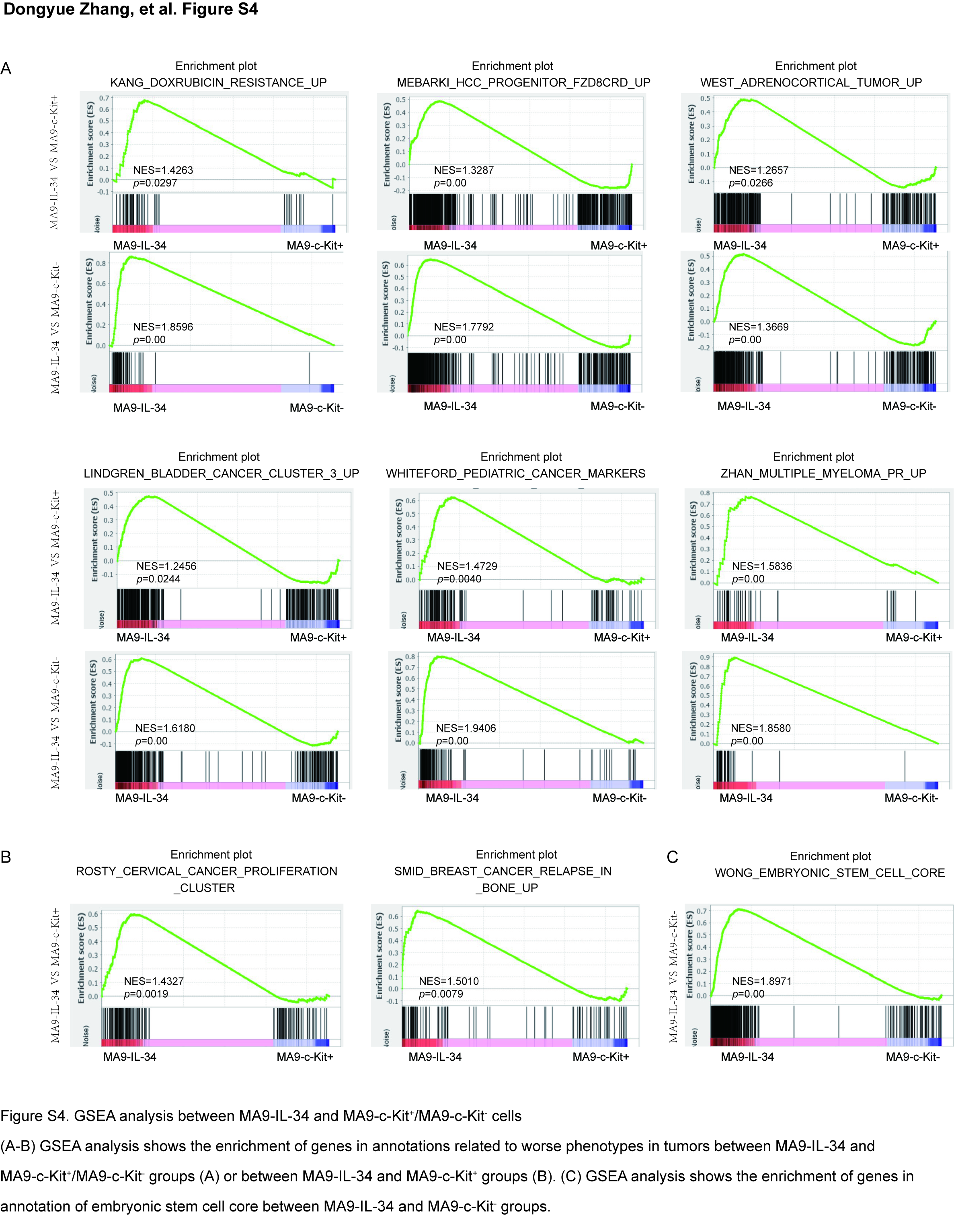

Supplement: Supplementary file 4 — Figure S4 [file 41419_2023_5822_MOESM4_ESM.tif]

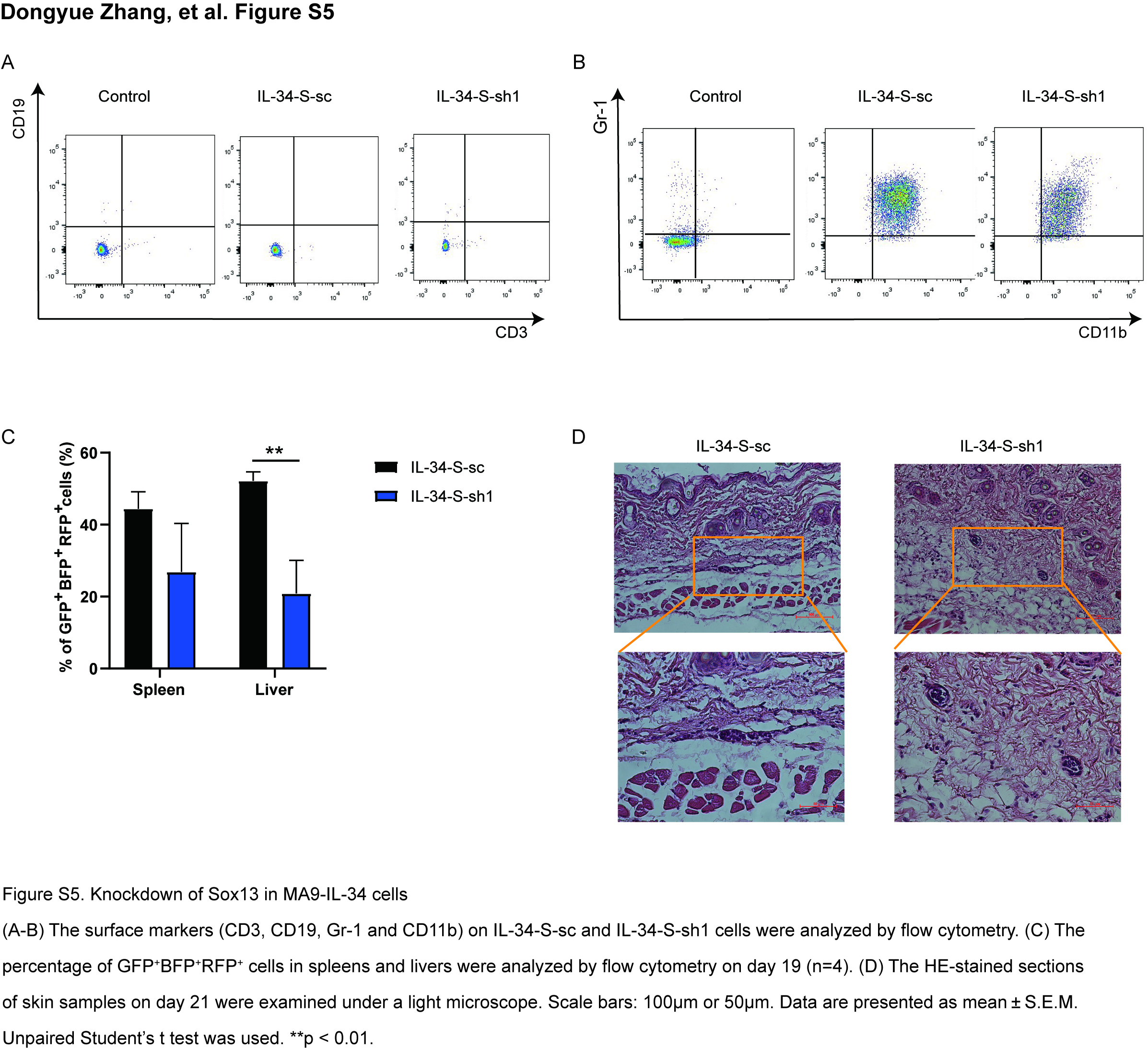

Supplement: Supplementary file 5 — Figure S5 [file 41419_2023_5822_MOESM5_ESM.tif]

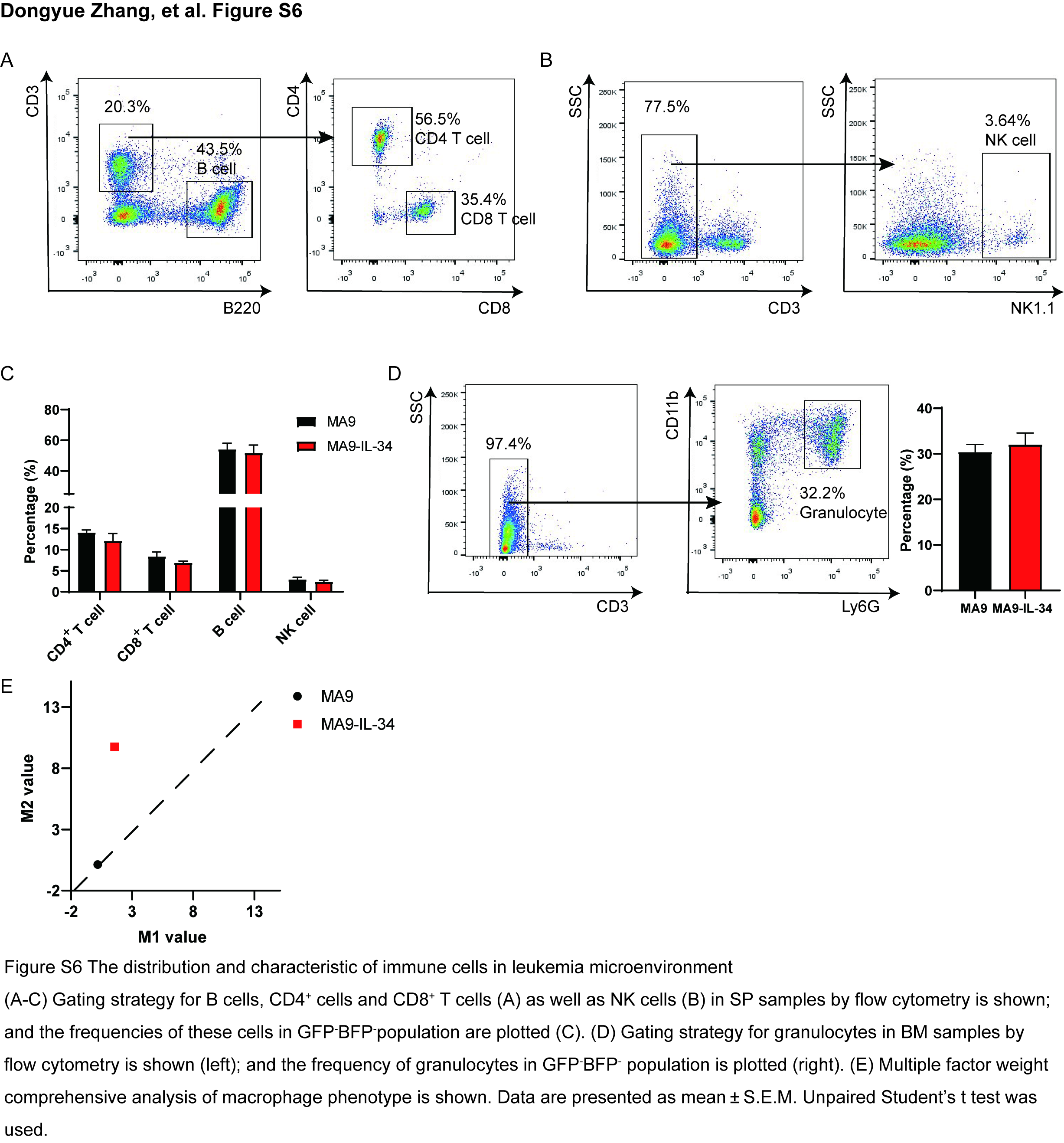

Supplement: Supplementary file 6 — Figure S6 [file 41419_2023_5822_MOESM6_ESM.tif]

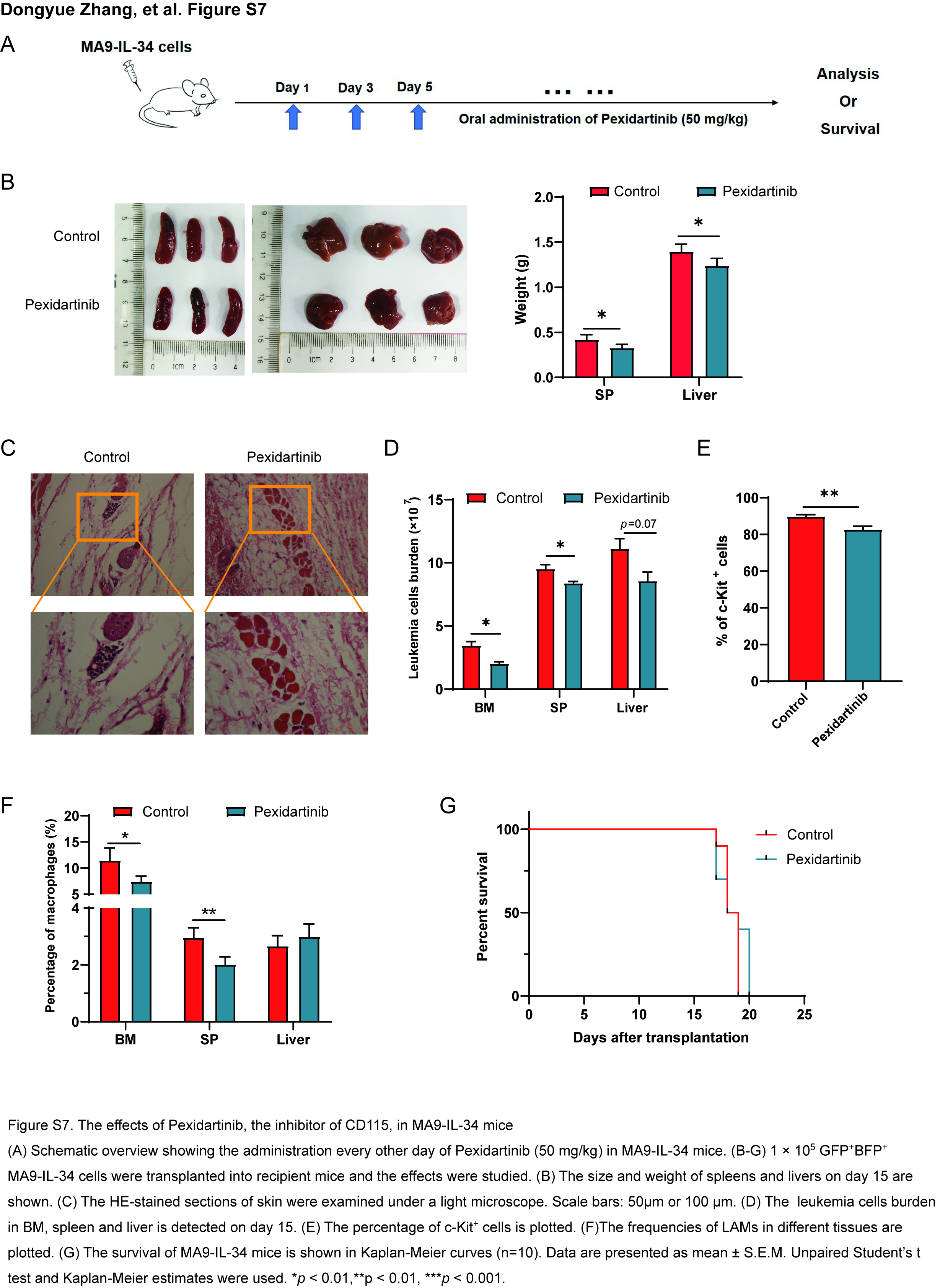

Supplement: Supplementary file 7 — Figure S7 [file 41419_2023_5822_MOESM7_ESM.tif]
