# Supplementary material for: Sox13 and M2-like leukemia-associated macrophages contribute to endogenous IL-34 caused accelerated progression of acute myeloid leukemia
Source: Cell Death Dis. 2023 May 6;14(5):308. doi: 10.1038/s41419-023-05822-z (PMC10164149; doi:10.1038/s41419-023-05822-z)
Supplement: Supplementary file 8 — Supplemental materials and methods [file 41419_2023_5822_MOESM8_ESM.docx]

**Materials and Methods**

**Antibodies and reagents**

Antibodies against mouse CD3 (17A2, PerCP-Cy5.5), CD19 (1D3, APC), NK1.1 (PK136, PE-Cy7), Ki67 (PE), BrdU (APC) and c-kit (2B8, PE-Cy7) were purchased from BD (USA). Antibodies against mouse Annexin V (APC), CD11b (M1/70, PerCP-Cy5.5), CD115 (AFS98, PE), Gr-1 (RB6-8C5, PE-Cy7), CD4 (GK1.5, PE-Cy7), CD8 (53-6.7, PE), B220 (RA3-6B2, PE), Ly6G (1A8, APC-Cy7) and CD45.2 (104, PE) were products of BioLegend (USA). Antibody against mouse F4/80 (BM8, APC) was purchased from Invitrogen (USA). Monoclonal antibody against GAPDH (17#2118) was from Cell Signaling Technology (USA). Polyclonal antibodies against IL-34 (ab101443) were purchased from Abcam (USA).

M3434 was purchased from Stem Cell Technologies (Canada). Fetal bovine serum (FBS), OPTI-MEM, sodium pyruvate, L-glutamine, penicillin/streptomycin, nonessential amino acids, and trypsin were purchased from Gibco (USA).

**Mice**

C57BL/6J and C57B6.SJL mice were provided by the Animal Center of the Institute of Hematology and Blood Diseases Hospital, CAMS & PUMC. Female mice (seven to eight weeks old) were used and maintained in specific pathogen-free (SPF)-certified facilities. All experiments were approved by the Animal Care and Use Committee at the institution.

**AML mouse model with high-level IL-34**

The MLL-AF9 induced mouse AML model was first described in 2006 [1] and widely used in leukemia study in the literature [2-4]. Briefly, Lin^-^ cells from normal C57B6.SJL (CD45.1) mice were sorted, infected with MSCV-MLL-AF9-GFP retrovirus in vitro and transplanted into healthy recipient mice by tail intravenous injection. All mice would suffer AML. Then, GFP^+^ leukemia cells (CD45.1) were sorted from leukemia mice and infected with blank (pMSCV-PGK-BFP) or IL-34 (pMSCV-IL-34-PGK-BFP) retrovirus. GFP^+^BFP^+^ cells were sorted by flow cytometry and transplanted into C57BL/6J (CD45.2) mice to establish the AML models.

**Analysis of AML progression**

In most experiments, 1×10^5^ MA9 or MA9-IL-34 cells were transplanted into C57BL/6J mice. In Sox13 KD experiments, 1×10^4^ IL-34-S-sc and IL-34-S-sh1 cells were transplanted. The percentage of AML cells in peripheral blood (PB), bone marrow (BM) and spleen was monitored by flow cytometry. The survival of mice was recorded. In some experiments, the spleens and livers were obtained and weighed. The tissues including BM, spleen, liver, kidney and skin were obtained and fixed following standard protocols. The tissue sections were undergone standard HE staining.

**Western blot**

Standard protocols were followed as previously describe. Briefly, cells were lysed in 1 × RIPA lysis buffer (Cell Signaling Technology, USA) containing phenylmethylsulfonyl fluoride (PMSF). The protein concentration was determined by BCA Protein Assay Kit (Thermo Scientific, USA). The samples were subjected to 10% SDS-PAGE for electrophoresis and transferred to a polyvinylidene difluoride membrane. Mouse monoclonal antibody against IL-34 or GAPDH was used as primary antibody. Goat-anti-mouse-HRP polyclonal antibodies were used as secondary antibodies. The proteins were chemiluminescent visualized using ChemiDoc (General Electric Company, USA). Data were acquired by gray value analysis (ImageJ).

***In vitro* phagocytosis assay**

Macrophages were sorted from BM by flow cytometry. FITC-labeled latex beads (2μm, Sigma-Aldrich) were added to 1×10^5^ macrophages in 500 μl RPMI 1640 complete medium and incubated for 30 min at 37°C. The samples were washed twice with cold PBS and resuspended in 300 μl PBS for flow cytometry.

**Pexidartinib treatment**

The CD115 inhibitor Pexidartinib (MedChemExpress, USA) was used to block the CD115 signal. Mice were transplanted with 1 × 10^5^ MA9-IL-34 cells on day 0. The mice were administered Pexidartinib (50 mg/kg) every other day by oral gavage from day 0 to the end of their lives.

**Flow cytometry analysis and cell sorting**

CantoII ﬂow cytometer and FACS AriaI II (BD Biosciences, USA) were used for FACS analysis and cell sorting, respectively. All experiments were completed following standard protocols. Data analysis was carried out using Diva (BD Biosciences, USA) and Flow JoV_10 (Tree Star, USA) software.

**RNA extraction and quantitative reverse-transcription polymerase chain reaction (qRT-PCR)**

Total RNA was extracted using TRIzol reagent (Life Technologies, USA) and reverse transcribed using Transcript All-in-one First-Strand cDNA Synthesis SuperMix (TransGen Biotech, China) following the manufacturers’ protocols. SYBR Green Kit (TaKaRa, China) was used for qRT-PCR experiments, which were performed on 0.1-QuantStudio 5 or 0.2-QuantStudio 5 (Thermo Fisher Scientific, USA). The expression level of target genes was expressed as the RQ value calculated by the ^ΔΔ^Ct method [^ΔΔ^Ct= (Ct_TARGET_−Ct_GAPDH_) _sample_− (Ct_TARGET_−Ct_GAPDH_) _calibrator_].

**References:**

[1] Somervaille TC, Cleary ML. Identification and characterization of leukemia stem cells in murine MLL-AF9 acute myeloid leukemia. Cancer Cell 2006, 10: 257-68.

[2] Cheng H, Hao S, Liu Y, Pang Y, Ma S, Dong F*, et al.* Leukemic marrow infiltration reveals a novel role for Egr3 as a potent inhibitor of normal hematopoietic stem cell proliferation. Blood 2015, 126: 1302-13.

[3] Feng W, Yang X, Wang L, Wang R, Yang F, Wang H, *et al*. P2X7 promotes the progression of MLL-AF9 induced acute myeloid leukemia by upregulation of Pbx3. Haematologica 2021, 106: 1278-89.

[4] Yang X, Feng W, Wang R, Yang F, Wang L, Chen S*, et al.* Repolarizing heterogeneous leukemia-associated macrophages with more M1 characteristics eliminates their pro-leukemic effects. Oncoimmunology 2018, 7: e1412910.
