# Supplementary material for: Sox13 and M2-like leukemia-associated macrophages contribute to endogenous IL-34 caused accelerated progression of acute myeloid leukemia
Source: Cell Death Dis. 2023 May 6;14(5):308. doi: 10.1038/s41419-023-05822-z (PMC10164149; doi:10.1038/s41419-023-05822-z)
Supplement: Supplementary file 9 — Supplemental table [file 41419_2023_5822_MOESM9_ESM.docx]

**Table 1. List of the primers used in qRT-PCR in this study**

| Gene | Forward (5’-3’) | Reverse (3’-5’) |
| --- | --- | --- |
| *mGapdh* | ACTCCACTCACGGCAAATTCAAC | GACACCAGTAGACTCCACGACAT |
| *hIL-34* | GCCGACTTCAGTACATGAAACACT | CCCTCGTAAGGCACACTGATC |
| *mIkzf2* | ATGATGACCGCCAGTCCTACC | GCCCTTATCTGTTCTCCTTCTCCAT |
| *mFgl2* | CTGACCAAGAGTAAGGAA | TTGTAGTTACCGATGTGTA |
| *mSox13* | GAACAGCAGCCACATCAAGAGAC | TCCACGACACAGGTACGCTTAG |
| *mProm1* | CCTCTCCCTCCTGGTGATTTGT | GCCTTGTTCTTGGTGTTGGTGTA |
| *mGfi1* | GACCCTTTGCGTGCGAGATG | TGTCCGAGTGAATGAGCAGATGT |
| *Cxcl11* | AGCTGCTCAAGGCTTCCTTA | GTGAACGTTGCCCATCATCATC |
| *IL-1b* | TGCCACCTTTTGACAGTGAT | TGTCCTCATCCTGGAAGGTC |
| *IL-6* | CCGCTATGAAGTTCCTCTCTGC | ATCCTCTGTGAAGTCTCCTCTCC |
| *Tnf-a* | AAGCCTGTAGCCCACGTCGTA | GGCACCACTAGTTGGTTGTCTTTG |
| *IL-12b* | ATGTGGAATGGCGTCTCTGTCT | TGGGCGGGTCTGGTTTGA |
| *iNOS* | CAGCGGAGTGACGGCAAAC | AGACCAGAGGCAGCACATCAA |
| *Cd206* | CCTGAACAGCAACTTGACCA | GCAATGGCCATAGAAAGGAA |
| *Arg1* | CAACCAGCTCTGGGAATCTG | AATCGGCCTTTTCTTCCTTC |
| *Ccl17* | TGCTTCTGGGGACTTTTCTG | TGGCCTTCTTCACATGTTTG |
| *Il-10* | CCAGAGCCACATGCTCCTA | AGGGGAGAAATCGATGACAG |
| *Mmp9* | TGAGTCCGGCAGACAATCCT | CCCTGGATCTCAGCAATAGCA |
